# Rhino-Orbito-Cerebral Mucormycosis: A Challenging Case

**DOI:** 10.7759/cureus.44768

**Published:** 2023-09-06

**Authors:** Awatif El Hakkouni, Sara Harrar, Abdelhamid Hachimi, Mostafa Mezouari, Redouane Moutaj

**Affiliations:** 1 Parasitology-Mycology Laboratory, Mohammed VI University Hospital, Marrakech, MAR; 2 Critical Care Medicine, Mohammed VI University Hospital, Marrakech, MAR; 3 Parasitology-Mycology Laboratory, Ibn Sina Military Hospital, Marrakech, MAR

**Keywords:** surgical debridement, liposomal amphotericin b, ketoacidosis, angio invasive fungi, diabetes mellitus, rhino-orbito-cerebral mucormycosis, mucormycosis, mucorales

## Abstract

Mucormycosis is a rare opportunistic fungal infection caused by *Mucorales* and associated with high mortality rates. Rhino-orbito-cerebral localization usually occurs in individuals with uncontrolled diabetes mellitus. We report the case of a 41-year-old male, with previously undiagnosed diabetes, who presented with unilateral facial extensive black eschar and signs of diabetic ketoacidosis. Cerebral magnetic resonance imaging showed left pansinusitis, left craniofacial edematous infiltrate, and left proptosis. A left internal temporal abscess was identified at an early pre-suppurative stage. Magnetic resonance angiography revealed total occlusion of the left intracranial internal carotid artery. A histopathological study of nasal mucosa biopsy suggested mucormycosis. According to the clinical presentation and the radiological and histopathological findings, rhino-orbito-cerebral mucormycosis was presumed. Culture of nasal, ocular, and skin lesion specimens grew *Rhizomucor* *sp*. and confirmed the diagnosis. The patient was treated with systemic liposomal amphotericin B. He died of multiple organ failure before surgical debridement was possible as he was in critical condition requiring stabilization before surgical treatment.

## Introduction

Mucormycosis is a deadly emerging infection, caused by fungi belonging to the order *Mucorales* [1,2]. These opportunistic ubiquitous fungi can enter a vulnerable host by inhalation of spores, consumption of contaminated food, or after skin disruption. They are potentially angio- invasive, leading to vascular thrombosis and tissular necrosis [3]. It is worth noting that there has been an increase in mucormycosis cases in recent decades, owing primarily to an increase in the number of immunocompromised patients [4]. This difficult-to-manage infection has also gained public relevance in some countries during the coronavirus disease 2019 (COVID-19) pandemic, mainly in India. Hyperglycemia and steroid use were among the significant risk factors for mucormycosis in patients infected with COVID-19. Eighty percent of cases had pre-existing diabetes mellitus, while 14.9% also had concurrent diabetic ketoacidosis. In 76.3% of cases, intake of corticosteroids was documented in the management of COVID-19. COVID-19-associated mucormycosis had a mortality rate of 30.7% [5]. Hence, the recommendation is to control hyperglycemia and adopt judicious use of corticosteroids to reduce the mortality of COVID-19-associated mucormycosis [5].

Rhino-orbito-cerebral mucormycosis (ROCM) is more frequently recognized in poorly controlled diabetes mellitus and associated with high mortality rates above 50% [6]. Herein, we report a rare case of ROCM in a 41-year-old man with previously undiagnosed diabetes mellitus.

## Case presentation

**Figure 1:**
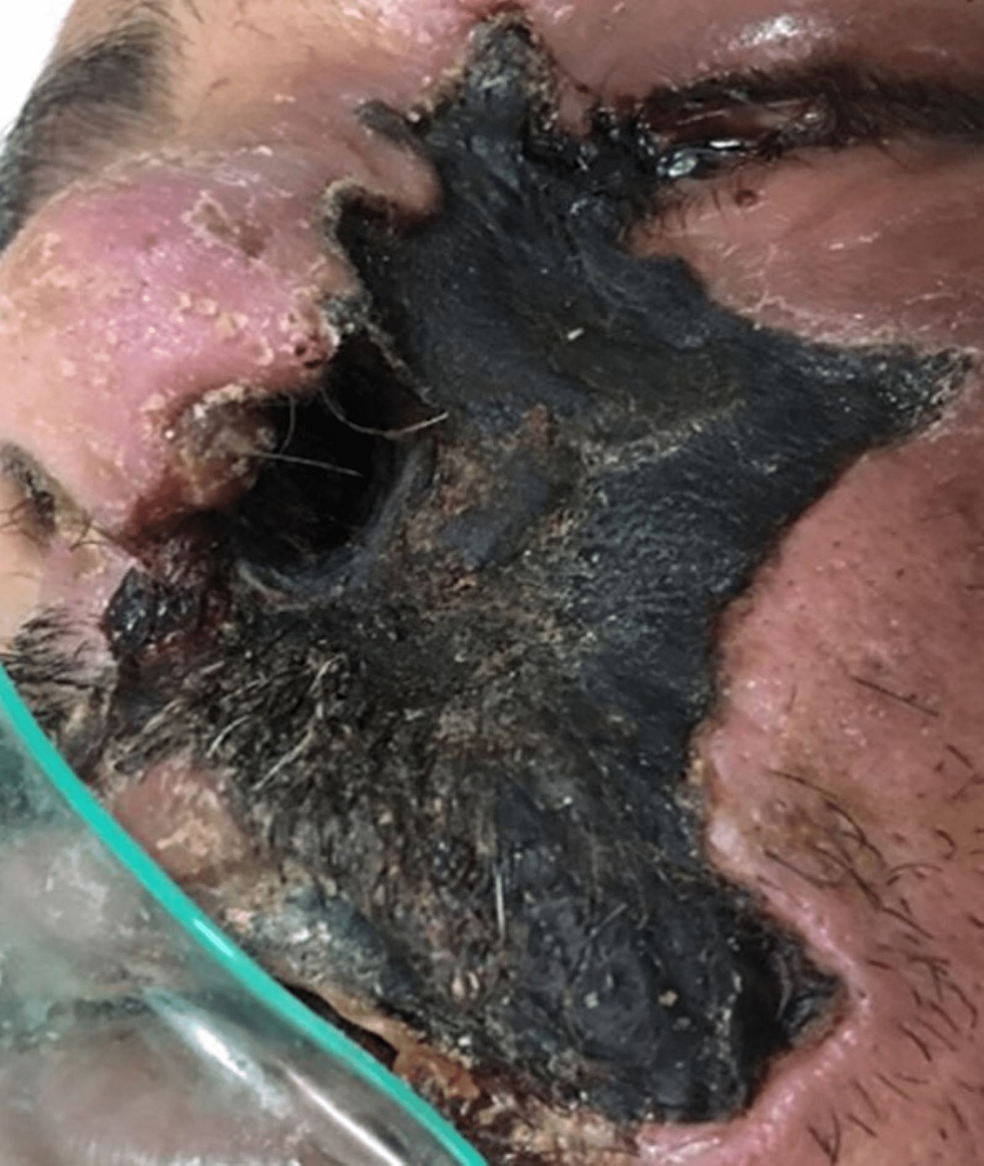
Black eschar of the nasal mucosa extending to the upper lip, oral cavity, left cheek, and orbit with proptosis and periorbital swelling.

A 41-year-old man was referred to the intensive care unit (ICU) with extensive necrotic facial cellulitis and widespread edema. Nasal mucosa's black eschar had spread to the left cheek, the left orbit, the upper lip, and the oral cavity (Figure 1), evolving toward periorbital swelling along with progressive loss of vision. The past medical history was unremarkable with no steroid intake. At admission, he was conscious and hypertensive (blood pressure: 150/60 mmHg) with high-grade fever (39°C) and SpO_2_ at 93%. The patient presented signs of diabetic ketoacidosis (hyperglycemia, ketonuria, a low level of bicarbonates in the blood: 10.86 mmol/L, increased HbA1C: 17%). Other biologic abnormalities included anemia (hemoglobin: 86 g/L) elevated white blood cells (27970/mm^3^ without blasts), elevated C-reactive protein (371 mg/L), creatinine (144 µmol/L), and hyperkalemia (6.9 mmol/L). Serum albumin was low (1.9 g/dL), and both gamma glutamyl transferase and lactate dehydrogenase levels were high. Serological tests for viral Hepatitis B, C and HIV 1,2 were negative.

**Figure 2:**
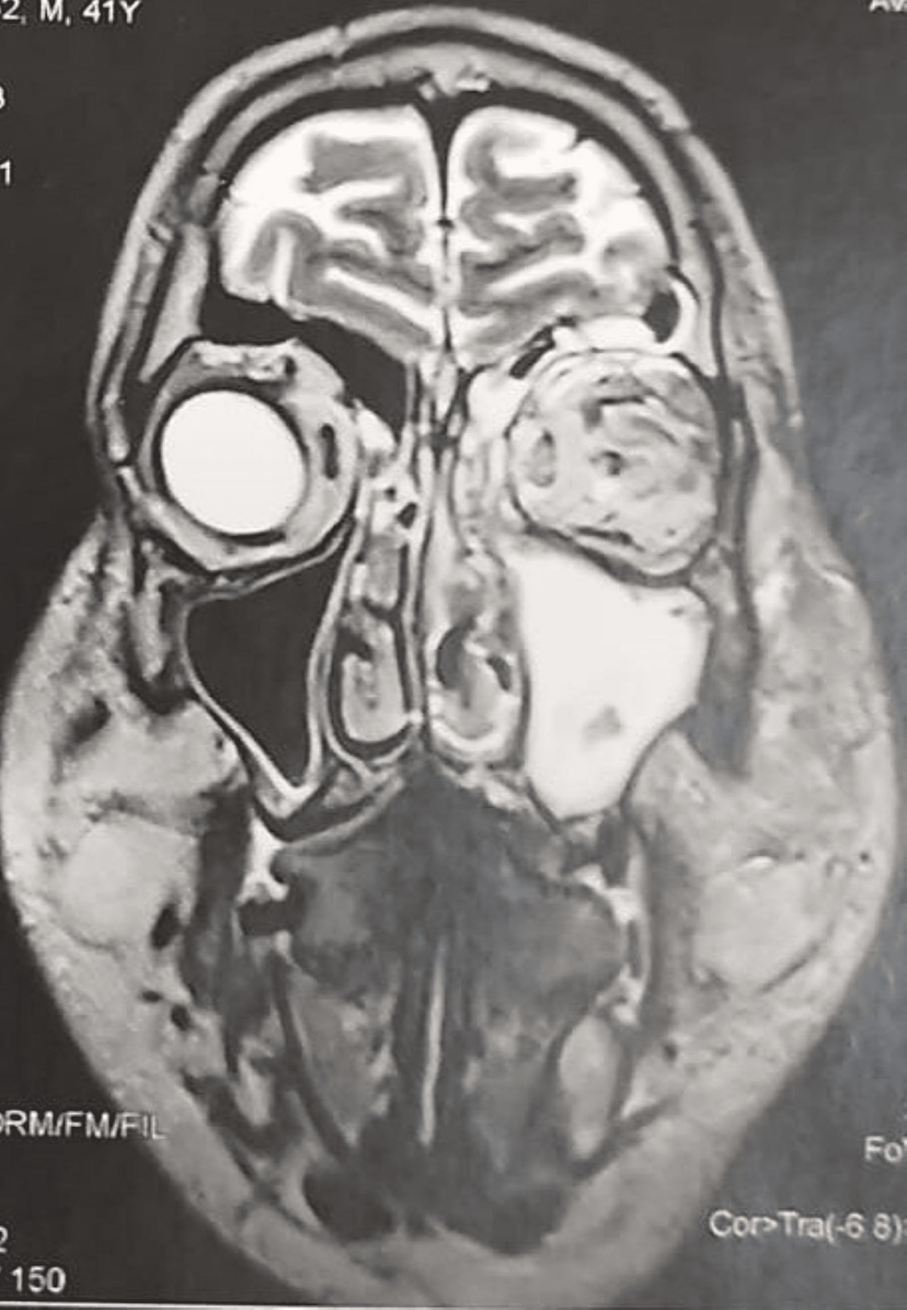
Coronal MRI showing left pansinusitis associated with left craniofacial and orbital edematous infiltrate.

**Figure 3:**
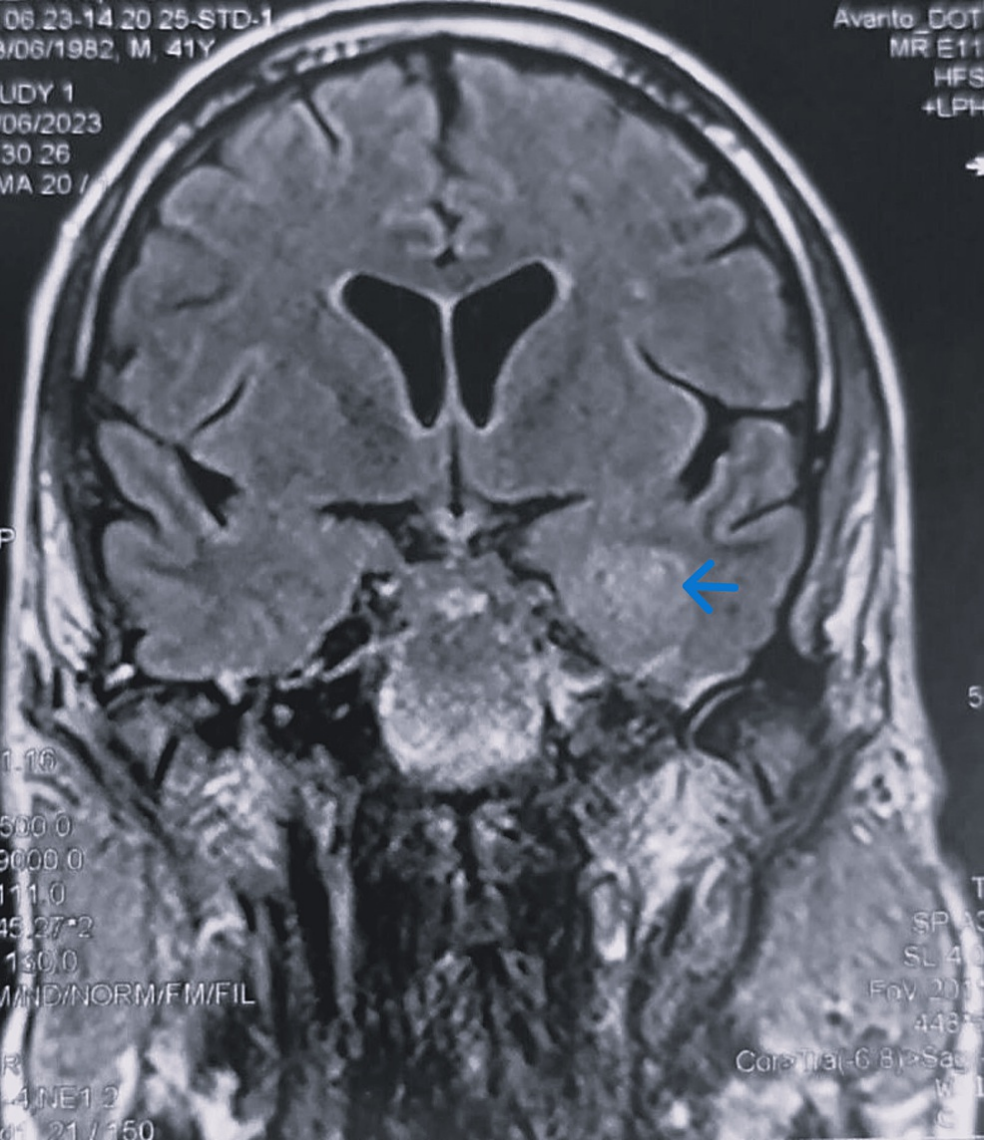
Coronal MRI showing an abscess at an early pre-suppurative stage in the left internal temporal region.

**Figure 4:**
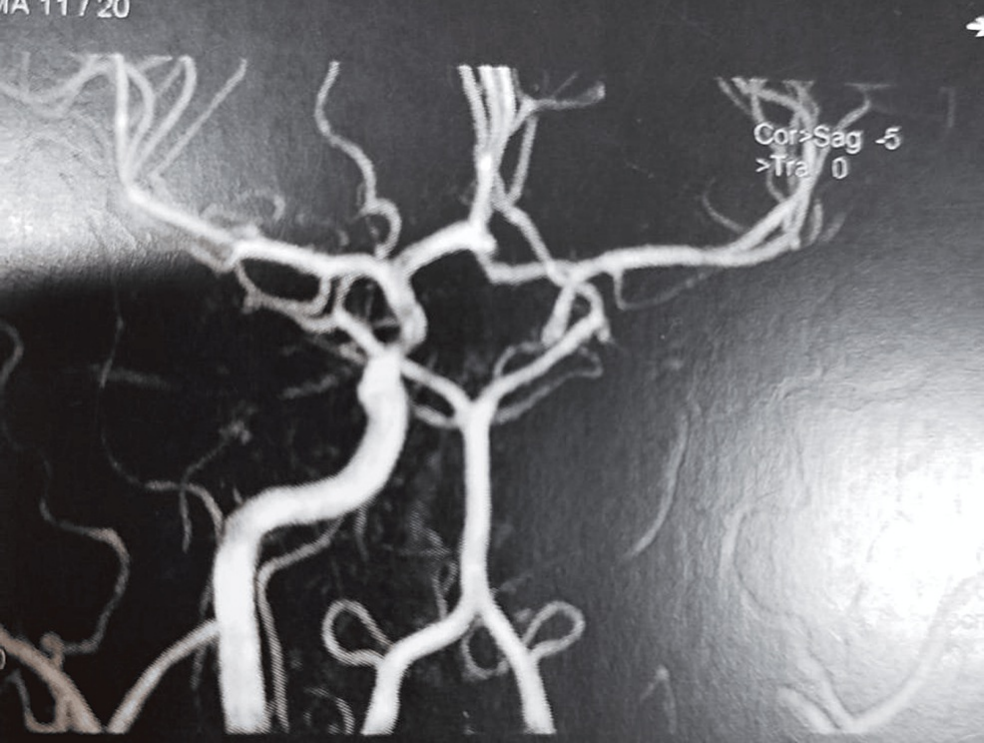
MRA showing total occlusion of the left intracranial internal carotid artery. MRA: Magnetic resonance angiography

Cerebral magnetic resonance imaging (MRI) showed left pansinusitis, left craniofacial edematous infiltrate, and left proptosis (Figure 2). In the left internal temporal region, an abscess at an early pre-suppurative stage was visible (Figure 3). Magnetic resonance angiography (MRA) revealed total occlusion of the left intracranial internal carotid artery (Figure 4). 

Histopathological study of nasal mucosa biopsy revealed chronic pyogranulomatous inflammation with non-septate, non-pigmented broad ribbon-like hyphae suggesting mucormycosis.

**Figure 5:**
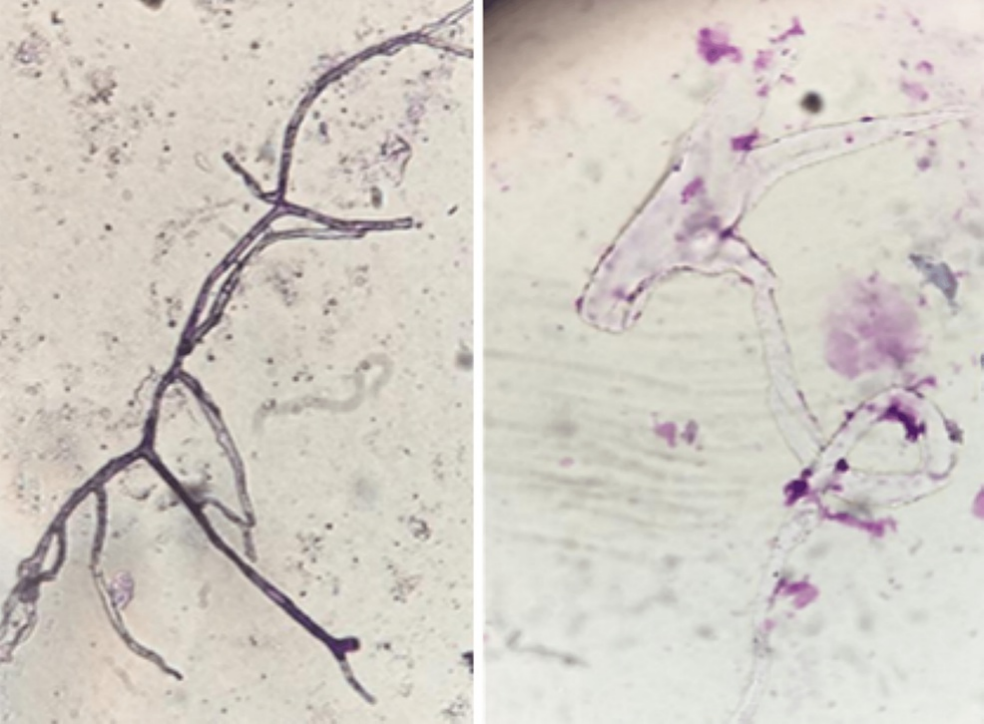
Direct microscopic examination of ocular discharge showing non-septate hyphae of Mucorales with irregular branching and 90°bifurcations. Left: 10% KOH mount. Right: MGG stain.

**Figure 6:**
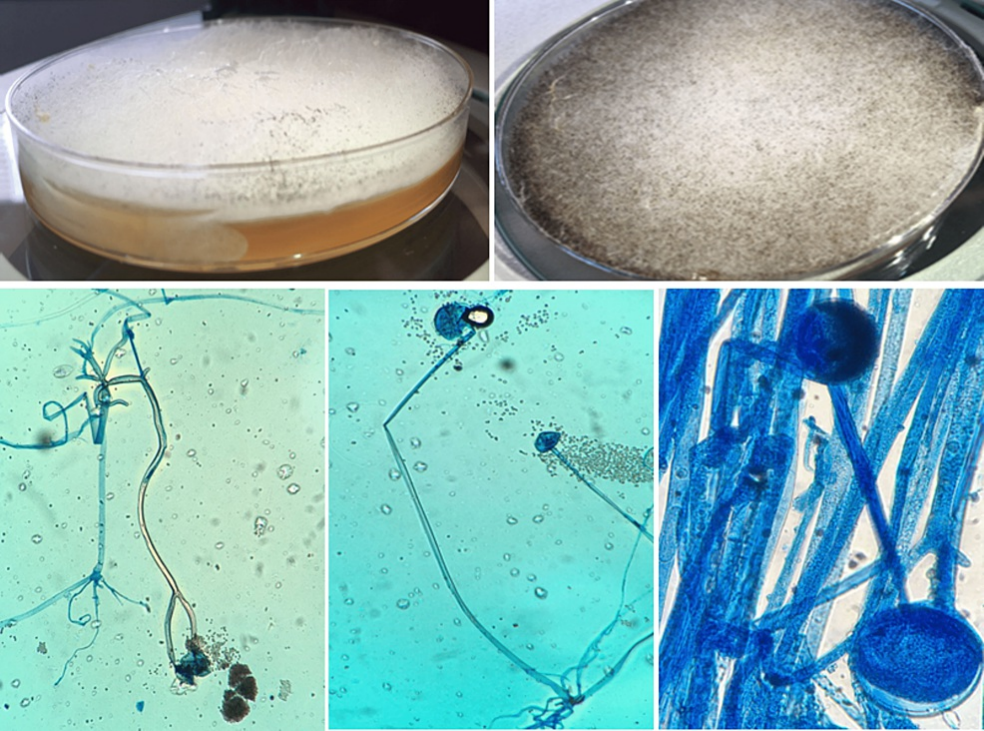
Grayish-white, cotton-like colonies of Mucorales on SDA (up). Branching sporangiophores with sporangia, rhizoïds, and poorly developed stolons, compatible with Rhizomucor sp (down).

Swabs of nasal and ocular discharge and the facial lesion were collected for culture on Sabouraud Dextrose Agar (SDA). Direct microscopic examination of the samples in 10% potassium hydroxide (KOH) mount and then May Grunwald Giemsa (MGG) staining found hyaline, non-septate irregular hyphae with branching at 90° angle, consistent with *Mucorales* infection (Figure 5). The culture grew *Rhizomucor* *sp.* (Figure 6) and confirmed the diagnosis of mucormycosis. Minimum Inhibitory concentrations were not available. 

During hospitalization in the ICU, among other measures of care, the patient received insulin therapy and blood transfusion. His blood glycemia was successfully controlled. He was treated with liposomal amphotericin B (lAMB) (5mg/kg/24H). He died due to multiple organ failure after four days of antifungal therapy, before surgical debridement was possible as he was in critical condition requiring stabilization prior to coordinated surgical multidisciplinary care.

## Discussion

Mucormycosis is a potentially fatal emerging infection, caused by fungi belonging to the order *Mucorales*, usually found in soil, and decomposing organic material. Eleven genera and thirty-eight distinct species of *Mucorales* are implicated in mucormycosis [1]. While *Rhizopus*, *Mucor*, and *Rhizomucor* are responsible for the majority of mucormycosis, other clinically important species include *Actinomucor*,* Apophysomyces*, *Cunninghamella*, *Lichtheimia*, *Saksenaea*, and *Syncephalastrum* [2].

Mucormycosis occurs mostly in hematology, solid organ transplant, and diabetic patients with hyperglycemia and ketoacidosis, although it can also develop in immunocompetent individuals after a trauma or burn [3]. Our patient had ketoacidosis, hyperglycemia, and elevated Hemoglobin A1C. Previously undiagnosed diabetes mellitus is an underlying condition known to be a major predisposing factor in mucormycosis [2].

ROCM is the most prevalent presentation in diabetic patients, especially those with poorly controlled diabetes mellitus [2,3]. Seventy percent of patients with ROCM are found to have underlying diabetes mellitus [7]. Usually, ROCM first affects the paranasal sinuses before spreading to nearby regions with bone destruction and invasion of the orbit, eye, and brain [8,9]. Mucorales' tendency for angio-invasion and its capacity to spread along perivascular spaces, however, allow for the invasion of neighboring structures even through intact bony partitions. This deadly infection with fulminant progression results in fungal thrombi, extensive tissue necrosis, and systemic dissemination [7]. ROCM is the most frequent manifestation of mucormycosis where intracranial fungal thrombi can spread directly or via blood vessels [10]. Numerous mechanisms of pathogenicity have been identified, including deficient chemotaxis, phagocytosis, and high GRP-78 protein expression in diabetics, which stimulates angio-invasion [8,11].

Imaging is essential for figuring out the extent of disease and directing medical and surgical treatment. Thus, cranial computed tomography or MRI is strongly recommended to determine if sinusitis is present in diabetic patients with facial pain, signs of sinusitis, proptosis, ophthalmoplegia, or amaurosis [8]. In our case, cerebral MRI showed left pansinusitis with significant infiltration of left subcutaneous soft tissues extending to the left periorbital region and leading to proptosis. It also revealed cerebral extension with a left internal temporal abscess, and MRA found total occlusion of the left intracranial internal carotid artery. These radiological manifestations might be consistent with deep, aggressive extension of the visible facial necrotic process.

Early clinical recognition and a multidisciplinary approach are mandatory for the successful management of mucormycosis. However, where the clinical presentation or radiological characteristics are suggestive, urgent tissue histopathology and culture are required [2].

The fundamentals in diagnosing mucormycosis include direct microscopy, culture, and histopathology of various clinical samples. An essential diagnostic tool is tissue identification, which differentiates the presence of the fungus as a pathogen in the specimen from its presence as a contaminant in culture [4]. It can be difficult to diagnose mucormycosis on the basis of histomorphology, and the most common reason for erroneous morphological diagnosis is mistaking *Aspergillus spp*. for *Mucorales*, especially when fungus hyphae are distorted or scarce [8]. However, a requirement for identifying mucormycosis as a confirmed infection is still the presence of broad, aseptate, or pauci-septate hyphae with wide-angle branching in affected tissue and signs of tissue invasion [12]. In our case, histopathology of nasal mucosa biopsy suggested mucormycosis. The mycological study of nasal and ocular discharge as well as cutaneous swabs from the infection site was positive showing hyaline non-septate irregular hyphae with irregular branching. Culture grew *Rizomucor sp*. All clinical, radiological, and pathological findings were consistent with ROCM in a diabetes mellitus patient with ketoacidosis.

Except for amphotericin B (AMB), mucorales exhibit intrinsic resistance to the majority of antifungals used in clinical settings, giving medical professionals few therapeutic options [1]. The use of lAMB formulations over conventional ones (cAMB) is preferred because they allow to administer higher doses with fewer side effects and better tolerability [10]. The gold standard is high-dose lAMB (5-10 mg/kg per day iv), given for a minimum of 6 to 8 weeks, but cAMB (1 mg/kg per day iv) is used in many countries due to its availability and lower cost [9]. The European Medicine Agency and the FDA have both approved newer broad-spectrum azoles, including posacanazole and isavuconazole, for the treatment of mucormycosis as salvage therapy [9]. Itraconazole was utilized to treat some cases of mucormycosis caused by *Rhizopus arrhizus* following amphotericin B therapy. The minimal inhibitory concentration for itraconazole in this particular species is low [13].

Regular monitoring of the renal functions, electrolyte balance, and diabetes mellitus status is required. The two main issues that are often seen during intravenous AMB therapy and need to be dealt with daily are hypokalemia and acute kidney injury [10]. Prompt institution of systemic antifungal therapy at the right dose significantly improves mucormycosis outcomes without affecting the precision of tissue diagnosis or cultures. Also, rapid reversal of the underlying predisposing factors and correction of metabolic abnormalities, whenever possible, such as ketoacidosis and hyperglycemia in diabetes mellitus, may improve the disease's prognosis. Early extensive surgical debridement to reduce the fungal load is recommended in ROCM and should be repeated if needed, to achieve local control and reduce mortality [3,8]. Mortality rates of mucormycosis remain extremely high, despite advances in management. Cerebral involvement has a poor prognosis. It is associated with a mortality rate of 65-80% [6]. The international multi-center study of Cag et al. evaluated the prognosis of mucormycosis patients in ICUs and revealed that the mortality rate was 80% [6].

## Conclusions

Rhino-orbito-cerebral mucormycosis is a rare, fulminant, and invasive fungal infection, associated with significant morbidity and mortality in diabetic patients. This difficult-to-diagnose disease is also difficult to treat. Early diagnosis, prompt institution of lAMB therapy, and urgent aggressive and repetitive surgical debridement associated with control of underlying predisposing factors are mandatory for successful management and improved prognosis.
